# Implementation and certification of a heart failure clinical care program in a middle income country: impact in clinical outcomes after 2 years

**DOI:** 10.1186/cc14664

**Published:** 2015-09-28

**Authors:** Pedro Gabriel MB Silva, Antonio Baruzzi, Douglas Ribeiro, Flavio Brito, Giuliano Generoso, Jose Teixeira, Marcelo Jamus, Mariana Okada, Thiago Macedo, Valter Furlan

**Affiliations:** 1 Hospital Totalcor, Cerqueira Cesar, São Paulo, São Paulo, Brazil

## Introduction

Clinical care programs (CCP) that monitor and optimize care have the potential to improve outcomes; however, their real benefits are still controversial.

## Objective

This study aims to evaluate the hypothesis of benefits in clinical outcomes after 2 years of a CCP.

## Methods

Prospective study of consecutive patients hospitalized with HF in a Brazilian private cardiovascular center. Two groups were compared based on the time to CCP initiation: the historical group, compounded by patients from the 6 months prior to CCP (group 1); and the intervention group, compounded by patients admitted with diagnosis of HF from July 2012 until June 2014, the period when patients and staff were monitored on a daily basis by a case manager nurse and a medical leader which provided educational interventions. The CCP was certified by an international society in October 2012.

## Results

In a total of 2188 patients, the mean age was 69.3 years and 55.8% were male (Table 1). Evidence-based therapies at hospital discharge (ACEI/ARB and beta-blocker in eligible patients) showed no significant change (95.8% pre-CCP and 97.5% post-CCP; *p *= 0.12). The outcomes analyzed in groups 1 and 2, were, respectively: hospital readmissions due to HF within 30 days (13.9% vs. 9.1%; *p *= 0.008); length of stay (8.9 ± 7.9 days vs. 7.9 ± 5.6 days, *p *= 0.01); decompensation of HF by poor adherence (16.8% vs. 10.5%; *p *= 0.001); and in-hospital mortality (9% vs. 6.9%; *p *= 0.24).

**Table 1:** 

	Pre-CCP (historical group)	CCP (intervention group)
Number of patients	338	1850
Mean age	71 (±13.5)	69 (±11.2)
Male (%)	55% (95% CI: 50-60%)	56% (95% CI: 54-58%)
Mean EF (%)	37% (±13.3)	42% (±11.1)
Hemodynamic profile C (%)	5.65% (CI95%: 3-8%)	4.2% (95% CI: 3.4-5.2%)
Ischemic cardiopathy (%)	48.2% (CI95%: 43-53.5%)	58% (95% CI: 56-60%)
HFpEF (%)	37% (CI95%: 32-42%)	27% (95% CI: 25-29%)
Cardiorenal syndrome (%)	35% (CI95%: 30-40%)	33% (95% CI: 31-35%)
Infection (%)	23% (95% CI: 19-28%)	22% (95% CI: 20-24%)

## Conclusion

During the 2 years of the CCP there was a reduction of 1 day in the length of stay, and a lower frequency of hospitalizations by poor treatment adherence, and in readmissions in 30 days.

